# Unraveling bisphenol A pharmacokinetics using physiologically based pharmacokinetic modeling

**DOI:** 10.3389/fphar.2014.00292

**Published:** 2015-01-09

**Authors:** Xiaoxia Yang, Jeffrey W. Fisher

**Affiliations:** Division of Biochemical Toxicology, National Center for Toxicological Research, U.S. Food and Drug Administration, Jefferson, AR, USA

**Keywords:** bisphenol A, PBPK, pharmacokinetics, age-dependent pharmacokinetics, species extrapolation, dosimetry

## Abstract

Physiologically based pharmacokinetic (PBPK) models integrate both chemical- and system-specific information into a mathematical framework, offering a mechanistic approach to predict the internal dose metrics of a chemical and an ability to perform species and dose extrapolations. Bisphenol A (BPA), because of its ubiquitous presence in a variety of consumer products, has received a considerable amount of attention from the public and regulatory bodies. PBPK models using deuterated BPA were developed for immature and adult rats and non-human primates and for adult humans to understand better the dosimetry of BPA. The focus of the present paper is to provide a rationale for interpreting species- and age-related pharmacokinetics of BPA. Gastrointestinal tract metabolism was an important consideration to predict unconjugated BPA serum kinetic profiles in adult and immature rats and monkeys. Biliary excretion and enterohepatic recirculation of BPA conjugates (BPA-c) accounted for the slowed systemic clearance of BPA-c in rats. For monkeys, renal reabsorption was proposed as a mechanism influencing systemic clearance of BPA-c. The quantitative understanding of the processes driving the pharmacokinetics of BPA across different species and life stages using a computational modeling approach provides more confidence in the interpretation of human biomonitoring data and the extrapolation of experimental animal findings to humans.

## INTRODUCTION

Physiologically based pharmacokinetic (PBPK) modeling, with its integration of physiological and biochemical processes, provides a quantitative description of the pharmacokinetics of the parent chemical and its metabolite(s). Compared with empirical compartmental models that lack physiological and biochemical features, PBPK models allow for extrapolation of the kinetic behavior of chemicals across routes of exposure, doses and species. PBPK models help reduce the uncertainty associated with extrapolation of toxicity findings in laboratory animals to humans.

Bisphenol A (BPA), a high production volume industrial chemical, has been widely used for the manufacture of polycarbonate plastics and epoxy resins, both of which have broad applications in consumer products, medical devices, and the printing industry (Willhite et al., 2008). Over 90% of people in the US have detectable levels of BPA in their urine (Calafat et al., 2008; Koch et al., 2012). Several *in vitro* and *in vivo* toxicity assessments have been reported (Richter et al., 2007; NTP, 2008; WHO, 2011; Vandenberg et al., 2012).

To understand better the kinetics of BPA in laboratory animals and humans, a series of kinetic studies were conducted at the U.S. Food and Drug Administration’s National Center for Toxicological Research (NCTR) in rats, mice, and monkeys, including various life stages (Doerge et al., 2010a,b, 2011a). Single dose pharmacokinetic studies were undertaken using a relatively low dose (100 μg/kg) of deuterated BPA (d6-BPA). The use of d6-BPA provided a contaminant-free method to measure BPA in biological tissues and using a low dose of d6-BPA provided kinetic time course behaviors that would be expected to be relevant to typical aggregate environmental exposures of <1 μg/kg/day in humans (USFDA, 2010; Lakind and Naiman, 2011; USCDC, 2014). This manuscript explains our thinking for how we unraveled some of the route- and age-specific complexities of BPA kinetics described in the papers of Fisher et al. (2011) and Yang et al. (2013). This is important because other recent important PBPK models for BPA (Teeguarden et al., 2005; Edginton and Ritter, 2009; Mielke and Gundert-Remy, 2009) were carefully reviewed by EFSA (2014) and do not include presystemic metabolism in the gastrointestinal (GI) tract, a feature in our BPA models that helped to predict the extremely low oral systemic bioavailability of BPA. In addition, our rationale is explained for species extrapolation (scaling) of BPA model parameters in non-human primates to humans.

## INTRAVENOUS DOSING WITH d6-BPA

Armed with several high quality kinetic studies with d6-BPA in rats and monkeys (Doerge et al., 2010a,b), the initial PBPK model development focused on adult monkeys and rats dosed intravenously (i.v.) with d6-BPA. This was the least problematic route of administration and demonstrated classic flow-limited kinetic behavior for hepatic metabolism. The dose of d6-BPA was small; thus hepatic extraction (BPA conjugation) greatly exceeded the rate at which the blood supply perfused the liver with BPA. We knew that the rate of BPA conjugation was very fast because of the rapid disappearance of the parent BPA from serum and the prompt appearance of metabolites in serum (Doerge et al., 2010a,b). This occurred in every species tested at NCTR (mice, rats, and monkeys). The unconjugated d6-BPA kinetic behavior appeared uncomplicated (generally log-linear) after i.v. administration (Doerge et al., 2010a,b); however the d6-BPA conjugate behavior seemed more complex because of its prolonged terminal clearance phase.

A mass conservation equation, which implies that a compartment is well-stirred (uniform), was used to represent each model compartment. The solubility of BPA in the compartment (tissue/serum or blood partition coefficient), the concentration of BPA in blood or serum, and the perfusion rate of the compartment described the rate of BPA uptake and clearance from the compartment. An additional equation accounted for the metabolism of BPA in the liver. In our models, one of the simplifying assumptions was that the rate of formation of the metabolites was set equal to the rate of metabolism of d6-BPA. Hepatic BPA conjugation in rats was estimated using *in vitro* to *in vivo* extrapolation (IVIVE). A Michaelis–Menten equation, where the Michaelis constant was set equal to the reported *in vitro* K_m_ value determined with native hepatic microsomes from adult rats (Mazur et al., 2010), and the maximum hepatic reaction velocity was derived by scaling of the *in vitro* maximal velocity to predict rat serum time course data for d6-BPA (Doerge et al., 2010a). For monkeys, hepatic conjugation of BPA was determined by fitting serum d6-BPA time course data from i.v. dosing, using a first order metabolic constant (Doerge et al., 2010b).

**FIGURE 1:**
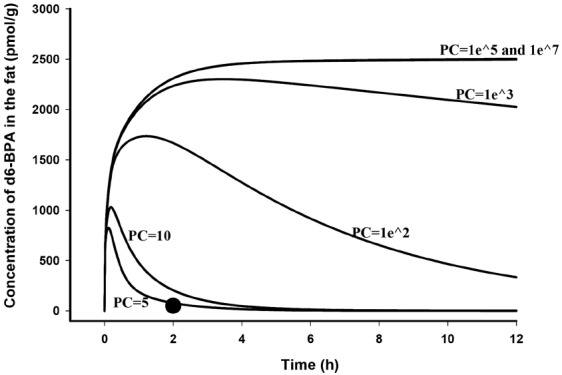
**Model simulated time course of d6-BPA concentrations (solid lines) in the fat of adult rats following i.v. dosing of 100 μg/kg d6-BPA with different fat to blood partition coefficients (PC) assumed, and measured d6-BPA concentration (●) in the fat at 2 h after dosing** (Doerge et al., 2011b).

**FIGURE 2:**
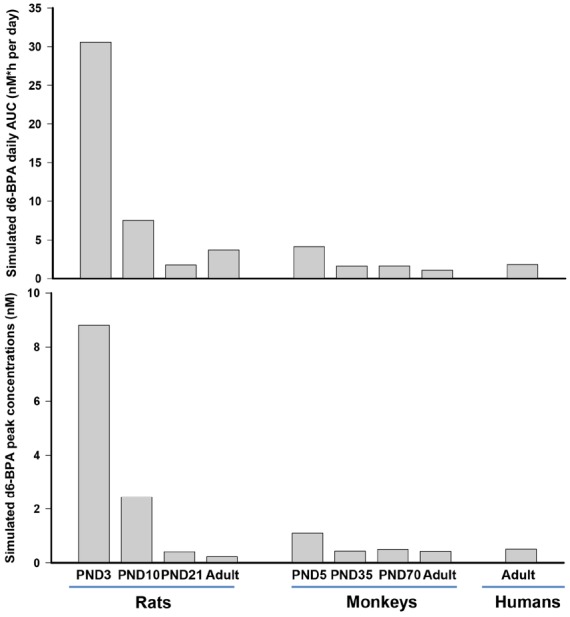
**Model simulated daily area under the serum concentration time curve (AUC) and peak concentration (C_max_) of d6-BPA at steady state in rats, monkeys, and humans.** Repeated daily oral dosing of 50 μg/kg of d6-BPA was simulated for 5–14 days to ensure that serum d6-BPA concentrations reached steady state levels.

The general success of the PBPK model to predict d6-BPA kinetics after i.v. administration in the rat and monkey (Figure 2 in Yang et al., 2013 and Figures 2 and 3 in Fisher et al., 2011) suggested that BPA kinetic behaviors in rats and monkeys were similar to many other well-metabolized chemicals that have been previously described using venous equilibration equations (Reddy et al., 2005). The notion that BPA is sequestered and retained in some part of the body, such as fat (Stahlhut et al., 2009), is highly improbable. The solubility of d6-BPA in fat relative to serum in rats was 5.0 at 2 h after i.v. dosing (Doerge et al., 2011b). Using vial equilibration and human tissues a fat:blood partition coefficient value of 3.3 was determined (Csanady et al., 2002). Similar to experimental findings, using structure-activity algorithms (Rodgers et al., 2005; Rodgers and Rowland, 2006; Schmitt, 2008), fat:blood and fat:plasma partition coefficients of BPA in humans were calculated to be 3.3 (Mielke et al., 2011; Partosch et al., 2013) and 8.3 (Edginton and Ritter, 2009). In addition, in adult mice the clearance of d6-BPA from fat paralleled the serum clearance of d6-BPA after i.v. dosing, signifying rapid equilibrium between blood and fat stores (Doerge et al., 2012). If d6-BPA were a highly fat soluble chemical, the expected behavior of d6-BPA in fat of adult rats would be much different (Figure 1) than what was observed 2 h after dosing (Doerge et al., 2011b). BPA is a moderately lipophilic chemical (log k_*ow*_ ∼ 3; Borrirukwisitsak et al., 2012) that equilibrates with tissues (diffuses into and out of tissues) freely.

For monkeys and rats, experimental studies demonstrated that the primary conjugate was the BPA glucuronide (BPA-glu), with the sulfate accounting for <20% for monkeys and <5% for rats (Doerge et al., 2010b). In monkeys, radioactive BPA administered by i.v. injection was excreted principally in urine (74–82%), with <3% found in feces (Kurebayashi et al., 2002). In rats, only 6–8% of the i.v. administered radioactive BPA was recovered in urine, with a much larger fraction excreted in feces as unconjugated BPA (46–49%; Kurebayashi et al., 2003). These conjugates are not associated with receptor-mediated toxicity of BPA. A simple modeling approach using one (monkey) or three (rat) compartments was sufficient to represent the conjugates as total BPA conjugates (BPA-c). Two different approaches were used to describe the systemic clearance of BPA-c. For rats, biliary excretion and enterohepatic recirculation of BPA-c were included in the model because BPA-glu was identified as a potential substrate of rat MRP2 (Mazur et al., 2012), which is an efflux pump present at the canalicular pole of rat hepatocytes (Ito et al., 1997). Eighty seven percent of BPA-glu formed in the liver was found to be excreted into the bile of rats (Inoue et al., 2005). Sakamoto et al. (2002) reported the de-conjugation of BPA-glu in the terminal region of the small intestine by bacteria, and the reabsorption of the formed unconjugated BPA. To describe the excretion of BPA-c into the bile, a Michaelis–Menten equation was used, based on experimental findings with monoglucuronosyl bilirubin and MRP2 (Jemnitz et al., 2010) and BPA biliary excretion data reported by Inoue et al. (2005). The mathematical description of enterohepatic recirculation (biliary excretion, bacterial deconjugation, reabsorption, and fecal excretion) simply used non-physiological terms constrained to predict slowed systemic clearance of serum BPA and BPA-c (Doerge et al., 2010a), and the reported fecal excretion of BPA (Kurebayashi et al., 2003). Unraveling some of the complexities of the GI tract for i.v. administration of 100 μg/kg of d6-BPA resulted in model predictions (and assumptions) that BPA-c formed in the liver was predominantly excreted into the bile, with only a very small portion (less than 1%) entering the systemic circulation. The biliary excreted BPA-c was subject to deconjugation in the terminal region of the GI tract. The majority (90%) of the unconjugated BPA formed in the gut lumen by bacteria was predicted to enter the enterocyte, undergo conjugation, and move into systemic circulation as BPA-c. The reminder of the unconjugated BPA was reabsorbed from the enterocyte into the system or excreted into feces.

In non-human primates (Kurebayashi et al., 2002) and humans (Völkel et al., 2002), BPA is predominantly excreted in the urine as BPA-c, thus BPA-c was not expected to be excreted in bile. Therefore, only a minimal effort was undertaken to evaluate the role of biliary excretion of d6-BPA-c in monkeys. Nevertheless, in order to predict the slowed systemic clearance of d6-BPA-c in monkeys, it was necessary to assume that a small fraction of d6-BPA-c underwent renal reabsorption (Fisher et al., 2011). This process was described with a Michaelis–Menten equation, based on the reported affinity of HEK-MRP2 toward bilirubin bisglucuronosyl (Jemnitz et al., 2010) and a maximum velocity term fitted to serum d6-BPA-c concentrations.

For humans, renal reabsorption may also play a role in the kinetic behavior of BPA-c. BPA-glu was identified as a potential human MRP3 substrate (Mazur et al., 2012). Weak expression of MRP3 has been observed in human kidney cell lines (Hilgendorf et al., 2007) and tissues located on the basolateral membranes (Scheffer et al., 2002). Renal reabsorption of BPA-c was later removed for human simulations (Fisher et al., 2011) because of the lack of direct support for recycling of BPA-c in the kidney.

## ORAL DOSING WITH d6-BPA

Because oral ingestion of BPA through the diet is the primary route of human exposure to BPA, NCTR conducted oral bolus gavage pharmacokinetic studies with d6-BPA in rats and monkeys using an aqueous vehicle (Doerge et al., 2010a,b). In Germany, oral dosing pharmacokinetic studies in humans were carried out with d16-BPA in a hard gelatin capsule (Völkel et al., 2002). These studies were used for the calibration of the orally dosed BPA in monkeys, rats, and humans, where the calibrated monkey model was extended to humans with the incorporation of human-specific physiological model parameters (Fisher et al., 2011). After oral bolus gavage of monkeys and rats with d6-BPA, unconjugated BPA only accounted for a very small fraction of the total dose based on non-compartmental analysis estimates. This was also true for humans ingesting d16-BPA, where only conjugated BPA was detectable in plasma. With such a low oral systemic bioavailability, our first exploratory evaluation using a PBPK model (calibrated for i.v. administration of d6-BPA) was to determine the influence of gastric emptying time and the rate of uptake of d6-BPA into the system from the gut lumen. Despite several combinatorial attempts to fit the data by adjusting the values for gastric emptying and the oral uptake rate of d6-BPA in the monkey and the rat, the model failed to predict the low serum concentrations of unconjugated BPA and its conjugated metabolites. We then speculated that a non-hepatic metabolic loss of d6-BPA was occurring based on the similar serum profiles of d6-BPA metabolites for both routes of administration.

From the literature, we found that the GI tract plays a major role in the metabolism of BPA. Inoue et al. (2003) reported BPA glucuronidation during its movement through the intestinal wall using a rat everted small intestine preparation. In other studies with rat intestinal microsomes, the rates of BPA glucuronidation were characterized (Ito et al., 2005; Mazur et al., 2010). Which UDP-glucuronosyltransferase (UGT) enzyme isoforms are responsible for BPA glucuronidation within rat intestine is unknown. Although there was no direct evidence for monkeys, GI tract BPA glucuronidation has been documented in human intestinal preparations (Mazur et al., 2010) and the human colon adenocarcinoma cell line (Audebert et al., 2011). Thus, we introduced a metabolism term for BPA in the GI tract for both rats and monkeys. For small experimental doses of BPA (100 μg/kg), presystemic metabolism of BPA within enterocytes was vital to obtain agreement between model predictions and observed serum time course profiles for d6-BPA in monkeys and rats after oral administration of d6-BPA. For rats, a composite first order term, representing the metabolism of BPA in the gut and the active transport of d6-BPA-c into the portal blood supply (see below), was derived by scaling an *in vitro* metabolic clearance rate (Ito et al., 2005) to describe the time course of serum d6-BPA and d6-BPA-c levels. For monkeys, gut metabolism of BPA was described using a first order metabolic term determined by visual fitting to serum d6-BPA concentration profiles after oral dosing (Doerge et al., 2010b). In humans, the scaled first order metabolic term derived from monkeys was used because of the lack of serum time course data for unconjugated BPA.

The unraveling of BPA kinetic behavior continued after GI tract metabolism of BPA was introduced into the model. To accommodate the rapid appearance of peak systemic levels of BPA-c, the fraction of BPA-c formed in enterocytes must be actively transported into systemic circulation. BPA-glu was identified as a potential substrate for the protein transporter MRP3 (Mazur et al., 2012). The presence of MRP3 on the basolateral membrane of enterocytes (Inokuchi et al., 2001; Rost et al., 2002) was hypothesized to account for the rapid active transport of BPA-c from enterocytes into the portal blood supply. Some evidence was available to support this assumption. Transport of BPA-glu from enterocytes into the serosal (blood) side was observed in the everted small intestine preparation of rats (Inoue et al., 2003). The influx of d6-BPA-c into the systemic circulation from enterocytes of monkeys and humans was described with a visually fitted first order term to predict serum levels of d6-BPA-c in monkeys after i.v. dosing. For rats, BPA-c produced from conjugation of BPA in enterocytes was assumed to be secreted instantaneously into the portal blood supply and this immediate transport process, along with the gut metabolism of BPA, was described with a composite first order constant.

## d6-BPA DOSING OF INFANTS

The concern about BPA exposure to sensitive sub-populations, such as infants and children, remains a research topic of interest. Thus, pharmacokinetic studies with d6-BPA were conducted in young monkeys and rats at NCTR (Doerge et al., 2010a,b). One very interesting contrast between immature rats and monkeys was that in rats, systemic d6-BPA exposure increased with decreasing age, but in monkeys, there was little difference in systemic d6-BPA exposure across all ages, [i.e., from postnatal day (PND) 5 to adults]. The adult PBPK models for oral administration of d6-BPA were modified to account for age-specific physiological model parameters and where literature information allowed, age-specific information for BPA was used. For example, Matsumoto et al. (2002) reported on the maturation of expression and activity toward BPA for UGT2B1, which is the predominant enzyme responsible for BPA glucuronidation in rat liver. Therefore, the metabolic constants representing the hepatic metabolism of BPA for young rats were initially set to a fraction of adult values to account for the reported maturity of hepatic UGT activity toward BPA and further adjusted to achieve agreement with serum d6-BPA and d6-BPA-c concentrations. In addition, Tomer et al. (2003) reported age-dependent changes for mRNA and protein levels of MRP2 in rat pup livers. As such, biliary excretion of BPA-c for pups was assumed to increase with age in accordance with the reported protein MRP2 levels.

Nevertheless, for the immature rat and monkey, the serum time-course kinetics provided the most important information. A fit-for-purpose approach was used where model predictions were accomplished by adjusting model parameters to obtain a consistent agreement between predictions and observations. Exploration of model parameters across different life stages suggested that the monkey and the rat display different patterns of maturation with respect to the metabolism of BPA. Immature monkeys and rats were predicted to have different maturation trajectories for hepatic metabolism of d6-BPA. The hepatic capacity to metabolize d6-BPA in young monkeys was predicted to be 8- to 15-fold less than the adult monkey, while for immature rats, in particular for PND10 and younger rats, the fold difference compared to adult rats was up to 282-fold. GI tract metabolism in the immature monkey was assumed to be much more active than in the immature rat, such that when combined with hepatic maturation, resulted in profound species differences (Figure 2) in pharmacokinetic profiles. Figure 2 shows model predicted peak concentration (C_max_) and the area under the serum concentration curve (AUC) of unconjugated BPA at steady state in rats, monkeys, and humans following repeated daily oral dosing of 50 μg/kg of BPA. As shown in Figure 2, the dose metrics of d6-BPA for PND10 and younger rats are greater than that in older rats, adult or infant monkeys, or adult humans.

Our speculation is that the infant monkey pharmacokinetic d6-BPA behavior is more representative of infants than using the immature rat pharmacokinetic d6-BPA behavior. The extrapolation of the newborn monkey model (PND5) to newborn humans resulted in steady-state average plasma BPA concentration of 0.002 μg/L for newborn humans after oral dosing of 1 μg/kg/day of BPA, which is 23 times less compared to the prediction of Edginton and Ritter (2009; 0.046 μg/L). As discussed above, for our modeling efforts, maturation of BPA metabolism was considered for both the liver and the GI tract. However, in the simulation of Edginton and Ritter (2009), only maturation of hepatic metabolism of BPA was considered for the extrapolation of the adult human model to infants.

## MODEL UNCERTAINTY

Professional judgment and interpretation of the literature played an important role in creating the BPA PBPK models. Gaps in data and knowledge exist for a quantitative understanding of the role of the GI tract in the disposition of BPA, e.g., conjugation of BPA in enterocytes and protein transporters for translocation of BPA and BPA metabolites, as well as bacterial de-conjugation of BPA metabolites (enterohepatic recirculation). Furthermore, no clinical pharmacokinetic studies exist for children or infants, so the model simulations are simply predictions using the best available extrapolation tools. The experimental animal kinetic data sets with d6-BPA help to reduce the uncertainty for the kinetic behavior of BPA, thus PBPK model development based on these data represent a solid foundation for age- and species-extrapolation.

Our reported sensitivity analyses for the BPA PBPK models (Fisher et al., 2011; Yang et al., 2013) suggest that both physiological and BPA specific model parameters are sensitive. The most sensitive model parameters in the monkey were related to the metabolism of BPA in the liver and oral uptake and metabolism of BPA in the GI tract. Experimental studies related to these sensitive model parameters could help refine the model parameter values.

## MODEL APPLICATIONS

The human BPA PBPK model performed well in comparison with experimental data (Völkel et al., 2002), with excellent fit to the time course of plasma BPA-c concentrations and predicted plasma BPA levels below the limit of detection (10 nM). One application of the current human PBPK model for BPA is to evaluate the expected serum concentrations of unconjugated BPA in the general adult population and for women of childbearing age (Teeguarden et al., 2013) using estimated BPA exposures from all sources of exposure (Lakind and Naiman, 2008). In the paper of Teeguarden et al. (2013), our model simulations predicted serum unconjugated BPA concentrations in the range of sub pM to pM for orally ingested BPA. These predictions were consistent with the calculated aglycone serum BPA concentrations reported in this paper (Teeguarden et al., 2013). For a small subset of measured aglycone serum BPA concentrations, the model predictions were much less, questioning the plausibility of these particular measurements in human serum.

In conclusion, our efforts to unveil quantitatively some of the complexities in the pharmacokinetics of BPA and its conjugates across species provide important information regarding dose-response toxicity studies with rats, including immature rats, and the interpretation of biomonitoring data. The characterization of BPA-c across species offers a better understanding of how the conjugates are formed, processed, and excreted. BPA dosimetry estimated in immature rats after oral dosing appears to over-predict BPA dosimetry in neonatal monkeys, suggesting that dose adjustment would be necessary if extrapolation of toxicity findings from immature rats to infants and children.

## AUTHOR CONTRIBUTIONS

Jeffrey W. Fisher and Xiaoxia Yang are listed as authors. They analyzed and interpreted the data; drafted the manuscript and revised it critically; final approved the version to be published; and agreed be accountable for all aspects of the work in ensuring that questions related to the accuracy or integrity of any part of the work are appropriately investigated and resolved.

### Conflict of Interest Statement

The authors declare that the research was conducted in the absence of any commercial or financial relationships that could be construed as a potential conflict of interest.

## Abbreviations

BPA: bisphenol A
BPA-c: bisphenol A conjugates
BPA-glu: bisphenol A glucuronide
d6-BPA: deuterated bisphenol A
GI: gastrointestinal
i.v.: intravenous
PBPK: physiologically based pharmacokinetic modeling
PND: postnatal day
UGT: UDP-glucuronosyltransferase.
